# La neurofibromatose de type 1

**DOI:** 10.11604/pamj.2013.15.98.3013

**Published:** 2013-07-13

**Authors:** Fadwa El Amrani, Nadia Ismaili

**Affiliations:** 1 Service de Dermatologie, CHU Ibn Sina, université Med V, Souissi, Rabat, Maroc

**Keywords:** Neurofibromatose, phacomatoses, autosomique dominante, neurofibromatosis, phacomatoses, autosomal dominant

## Image en médecine

La neurofibromatose de type 1(NF1) ou maladie de Von Recklinghausen est la plus fréquente des phacomatoses. C'est une affection autosomique dominante, où les mutations de novo concernent 50% des patients. Son diagnostic est clinique avec des critères définis. Les manifestations cutanées arrivent au premier plan avec les taches café au lait (TCL), les lentigines axillaires et inguinales et les neurofibromes cutanés. Les autres critères diagnostiques comprennent le gliome des voies optiques, les nodules de Lisch, la dysplasie sphénoïdale ou l'amincissement de la corticale des os longs ainsi qu'un parent du premier degré atteint de NF1. Aucun traitement curatif n'est disponible à ce jour. Nous rapportons l'observation d'une patiente de 32 ans, qui consulte pour une tumeur géante de la face latérale du sein gauche. L'examen dermatologique retrouvait les critères de la NF1: plus de 6 TCL de plus de 1,5 cm, des lentigines diffuses, des neurofibromes cutanés et un neurofibrome plexiforme géant prenant toute la partie latérale du sein gauche, mesurant environ 25 cm de grand axe. L'examen ophtalmologique a révélé de multiples nodules de Lisch bilatéraux. L'IRM cérébrale et les radiographies osseuses étaient sans anomalie. Une exérèse chirurgicale du neurofibrome plexiforme a été réalisée. Il n y a pas eu de récidive locale après deux ans de suivi.

**Figure 1:**
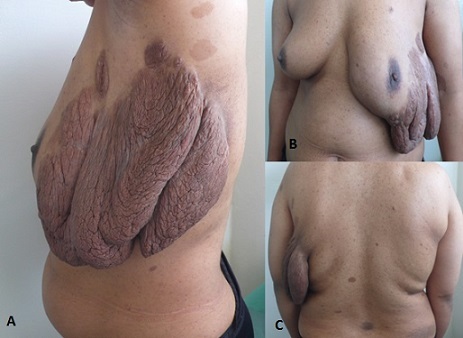
A: neurofibrome plexiforme géant prenant toute la face latérale du sein gauche B: neurofibrome plexiforme vu de face C: taches café au lait et lentigines diffuses

